# Survey of the total fatty acid and triacylglycerol composition and content of 30 duckweed species and cloning of a Δ6-desaturase responsible for the production of γ-linolenic and stearidonic acids in Lemna gibba

**DOI:** 10.1186/1471-2229-13-201

**Published:** 2013-12-05

**Authors:** Yiheng Yan, Jason Candreva, Hai Shi, Evan Ernst, Robert Martienssen, Jorg Schwender, John Shanklin

**Affiliations:** 1 Biosciences Department, BNL 463, 50 Bell Ave, Upton, NY 11973, USA; 2 Cold Spring Harbor Laboratory, 1 Bungtown Rd, Cold Spring Harbor, NY 11724, USA

**Keywords:** Desaturase, Fatty acid, Triacylglycerol, Lemnoideae, Duckweed, *Lemna*, *Wolffiela*, Renewable feedstock, Biofuel

## Abstract

**Background:**

Duckweeds, i.e., members of the Lemnoideae family, are amongst the smallest aquatic flowering plants. Their high growth rate, aquatic habit and suitability for bio-remediation make them strong candidates for biomass production. Duckweeds have been studied for their potential as feedstocks for bioethanol production; however, less is known about their ability to accumulate reduced carbon as fatty acids (FA) and oil.

**Results:**

Total FA profiles of thirty duckweed species were analysed to assess the natural diversity within the Lemnoideae. Total FA content varied between 4.6% and 14.2% of dry weight whereas triacylglycerol (TAG) levels varied between 0.02% and 0.15% of dry weight. Three FA, 16:0 (palmitic), 18:2Δ9,12 (Linoleic acid, or LN) and 18:3Δ9,12,15 (α-linolenic acid, or ALA) comprise more than 80% of total duckweed FA. Seven *Lemna* and two *Wolffiela* species also accumulate polyunsaturated FA containing Δ6-double bonds, i.e., GLA and SDA. Relative to total FA, TAG is enriched in saturated FA and deficient in polyunsaturated FA, and only five *Lemna* species accumulate Δ6-FA in their TAG. A putative Δ6-desaturase designated LgDes, with homology to a family of front-end Δ6-FA and Δ8-spingolipid desaturases, was identified in the assembled DNA sequence of *Lemna gibba*. Expression of a synthetic LgDes gene in *Nicotiana benthamiana* resulted in the accumulation of GLA and SDA, confirming it specifies a Δ6-desaturase.

**Conclusions:**

Total accumulation of FA varies three-fold across the 30 species of Lemnoideae surveyed. Nine species contain GLA and SDA which are synthesized by a Δ6 front-end desaturase, but FA composition is otherwise similar. TAG accumulates up to 0.15% of total dry weight, comparable to levels found in the leaves of terrestrial plants. Polyunsaturated FA is underrepresented in TAG, and the Δ6-FA GLA and SDA are found in the TAG of only five of the nine *Lemna* species that produce them. When present, GLA is enriched and SDA diminished relative to their abundance in the total FA pool.

## Background

Duckweeds are the smallest known aquatic flowering plants [1]. These monocotyledonous plants family are in the family Lemnoideae which contains five genera: *Lemna*, *Spirodela*, *Wolffia*, *Wolffiela* and *Landoltia*, encompassing more than 38 different species geographically distributed around the globe [2]. The morphology of different genera of duckweed differs greatly, ranging from the relatively complex structure of members of the genus *Spirodela* to the extremely reduced structures found in the genus *Wolffia*.

Duckweeds have been used for research since the 1960s [3] but genetic and molecular techniques have advanced more rapidly in other model systems. There is renewed interest in duckweed due to the high demand for renewable biomass. Many species of duckweed have rapid doubling times, as short as 48 hours in *Lemna aequinoctialis* and *Wolffia microscopica*[1], and certain duckweed species have the ability to grow on waste water [4]. Recent research has focused on the ability of duckweed to produce starch and protein, for instance, *Spirodela polyrhiza* has been shown to accumulate up to 20% dry weight as starch when grown on pig effluent [5]. These traits have made duckweed a desirable candidate for biomass production. *Lemna gibba* has also been engineered to produce monoclonal antibodies [6].

Both starch and oil are sinks for photosynthetically fixed carbon, but oil is highly reduced, having an energy density more than 2-fold that of starch. Plant oils have a wide variety of applications, including industrial feedstocks [7] biofuels and dietary supplements. Plant oil is generally harvested from seeds, but recent studies suggest that oil accumulation can be successfully engineered in vegetative tissue [8-12]. Although little data available on lipid composition and accumulation in duckweed, its short doubling time, substantial ability to store excess photosynthate as starch and ability to grow on wastewater make it a promising candidate to screen for potential vegetative oil production.

To explore the possibility of using duckweed for oil production, we conducted a survey of 30 different species spanning the overall diversity of this family with respect to FA and TAG abundance. This study also examines duckweed’s FA composition to identify strains of duckweed that accumulate specific FA that are of potential importance as industrial feedstocks or dietary supplements. The survey revealed that nine duckweed species accumulate Δ6-containing FA in the form of γ-linolenic acid (GLA) or stearidonic acid (SDA). While these fatty acids are rarely found in higher plants, they are found in borage [13], and *Echium*[14]. Δ6-containing FA are synthesized by desaturases that are commonly referred to as “front-end” because they introduce double bonds between the carboxyl group of the FA and an existing double bond [15] rather than between an existing double bond and the terminal methyl group as in the majority of desaturase enzymes. Front-end desaturases occur as C-terminal fusions with their electron donor, cytochrome b_5_ and contain a tri-partite histidine motif found in all desaturases [16], except that the first histidine in the third box is substituted for a glutamine [15]. Δ6 desaturase sequences cluster with a ubiquitous class of Δ8 sphingolipid long-chain-base (LCB) desaturases [17] preventing their functional designation to either class based on sequence alone. A detailed study of *Lemna gibba*, which contains both GLA and SDA, resulted in the identification of LgDes, a member of the Δ6/Δ8 desaturase family. The identity of LgDes as a Δ6 desaturase was confirmed by heterologous expression in *Nicotiana benthamiana*.

## Methods

### Plant materials and growth conditions

Duckweed lines were obtained from The Rutgers University Duckweed Stock Cooperative (http://www.ruduckweed.org), and were cultured in SH medium containing 1.6 g/L Schenk and Hildebrandt Basal Salt Mixture (Sigma) with 0.5% glucose (pH 5.7). Fronds were cultured in T-75 culture flasks containing 100 ml of the culture medium, at 22°C, under continuous fluorescent light (100 μE m^-2^ s^-1^).

### Biomass composition analysis

Duckweeds were harvested by filtration to remove excess media. Total dry weights, and lipid and metabolite contents were determined as previously described [18]. Briefly, duckweed tissue was homogenized in 3 mL methanol/water (4:3, v/v) with an Omni tissue grinder (Omni International, Merietta, GA) followed by the addition of 3.4 mL CHCl_3_ to create a biphasic solvent system (CHCl_3_/methanol/H_2_O, 8:4:3, v/v/v, [19]). The phases were separated by centrifugation at 3,000 xg at ambient temperature. The dry weight fractions of lipids (CHCl_3_ phase), free metabolites (methanol/water phase) and cell pellets (insoluble material) were obtained.

### TAG extraction

TAG was separated from the total lipid extract by thin layer chromatography (TLC). Approximately 25% of the total lipid extract was spotted on a silica gel plate along with an Arabidopsis seed oil (TAG) standard. The plate was then developed with hexane: diethyl ether: acetic acid (80:20:1,v/v/v). After development, lipids were visualized by incubation in iodine vapour. The mobility of TAG was identified by comparison to authentic standards and the silica zone containing TAG was collected by scraping the silica from the TLC plate.

### FA and TAG analysis

FA were converted to fatty acid methyl esters (FAMEs) by derivatization using boron trichloride methanol as previously reported [20,21] after addition of 100 μg of heptadecanoic acid as an internal standard. 4,4-dimethyloxazoline (DMOX) derivatives were generated by incubation of FAMEs with 2-amino-2-methyl-1-propanol under a nitrogen atmosphere at 190°C for 16 hours [21]. Pyrrolidone adducts were generated using a standard protocol [22]. To facilitate quantitation, 100 μg of heptadecanoic acid was added to each sample as an internal standard and the abundance of FA from TAG were determined by integrating the areas of each GC-MS peak relative to the internal standard. FA and TAG profiles were obtained with the use of a Hewlett Packard 6890 gas chromatograph equipped with an Agilent J&W DB 23 capillary column (30 m × 0.25 mm × 0.25 mm) and a model 5973 mass selective detector. Each sample was analysed using an injector temperature of 250°C and a program in which the oven was heated from 80-170°C at 20°C/min, and from 170-210°C at 5°C/min. The values are presented as mean percentages ± standard deviations, n = 3 or more.

### Nicotiana benthamiana transient expression system

*Nicotiana benthamiana* plants were grown for approximately 6 weeks in growth chambers under a 16/8 hour day/night cycle and approximately 160 μmol m^-2^ s^-1^ of light at 22°C. The transient expression protocol was based on the method of Schütze [23]. On the day before infiltration, plants were watered and, to reduce sample variability, upper and lower leaves were removed to leave 3 recently fully expanded leaves (approximately 10-12 cm in diameter) for infiltration. This procedure allows each leaf to be exposed to full light in the post-inoculation phase. A single colony of *Agrobacterium* containing a binary plasmid harbouring the target desaturase gene under the control of the 35S promoter [24] was cultured overnight at 30°C in 5 ml of LB medium containing appropriate antibiotics. Cells were collected by centrifugation at 4,000 ×g for 10 min, and resuspend in freshly prepared AS medium (10 mM MES-KOH, pH 5.6, 10 mM MgCl_2_, 150 μM acetosyringone) to an OD_600_ of 0.5 and incubated for 1.5 hr. at 22°C. This suspension was diluted 1:1 with an equivalent preparation of cells harbouring the p19 RNA silencing suppressor [25] and incubated for a further 1.5 hr at 22°C prior to infiltration with the use of a 1 ml needleless syringe pressed against the abaxial surface of the leaf. Approximately 8 discrete infiltrations were performed per leaf, with the position of treatment and control infiltrations assigned randomly. The perimeters of infiltrated areas and treatment codes were marked using ballpoint pen. Plants were returned to growth chambers under normal growing conditions and the leaves were harvested 4 days after infiltration for analysis.

## Results

### Total fatty acid content and composition

**Figure 1:**
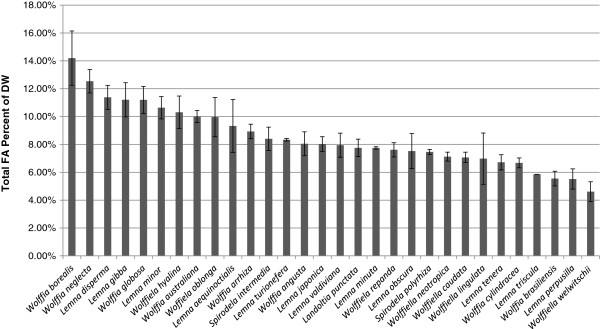
**Ranking of total FA content as a percent of dry weight for 30 duckweed species.** Means and standard deviations of three independent replicates are presented for each species.

Thirty species of duckweed were chosen to represent the widest available range of natural diversity within the Lemnoideae. Cultures were obtained from the Rutgers Duckweed Stock Cooperative and the provenance of each species is presented in Table 1. Cultures were grown on half-strength SH medium supplemented with 0.5% glucose for two weeks at which time 0.1 to 0.7 grams of fresh fronds were harvested for lipid analysis. To facilitate statistical analysis, three independent cultures were grown for each of the 30 species. Extracted lipids from each sample were converted to FA methyl esters (FAMEs) which were separated by capillary gas chromatography-coupled mass spectrometry (GC-MS) to obtain the composition of different FA species. The lipid content as a percentage of dry weight for each species is given in rank order in Figure 1. The FA content ranges from 4.6% for *Wolffiela welwischii* to 14.2% for *Wolffia borealis* with a median value of 8.0% for the 30 species. Fifty percent of the species fall between 6.9% and 10.1%. Standard deviations for individual species are typically <15% of the mean, and differences across the range of lipid content are highly significant (P <0.001 using student T-test).

**Table 1:** Providence of species used in this study

**Species**	**RDSC #**	**ID**	**Continent**	**Country**	**State/City/Notes**
*Landoltia punctata*	3	DWC014	South America	Venezuela	D. F., La Mariposa
*Lemna aequinoctialis*	62	8011	North America	USA	Oklahoma, Kay Co., Blackwell
*Lemna disperma*	320	7767	Australia	Western Australia	King River
*Lemna gibba G-3*	9	DWC130	North America	not known	not known
*Lemna japonica*	196	8693	Asia	Japan	Hokkaido, Setana
*Lemna minor*	376	DWC114	Europe	Switzerland	Ticino, Castel San Pietro
*Lemna minuta*	311	8430	Europe	United Kingdom	England, Cambridge
*Lemna obscura*	281	7143	North America	USA	Florida, Dade Co., Miami
*Lemna perpusilla*	274	8473	North America	USA	North Carolina, Johnston Co., Gees Cross Road
*Lemna tenera*	13	9024	Australia	Australia	Northern Territories, Nancar Billabong
*Lemna trisulca*	564	7413	Europe	Romania	Dobrogea, Maliuc
*Lemna turionifera*	125	8133	North America	USA	California, San Diego Co., Wohlford L.
*Lemna valdiviana*	198	8754	South America	Bolivia	Huatajata
*Spirodela intermedia*	151	7178	South America	Argentina	Buenos Aires, Buenos Aires
*Spirodela polyrhiza*	189	7498	North America	USA	North Carolina, Durham Co., Durham
*Wolffia angusta*	65	7274	Australia	New South Wales	Newcastle, Seaham
*Wolffia arrhiza*	52	7193	Africa	Uganda	Masaka
*Wolffia australiana*	82	DWC304	Australia	New South Wales	Singleton, Doughboy Hollow
*Wolffia borealis*	90	9147	North America	USA	Massachusetts, Franklin Co., Deerfield
*Wolffia brasiliensis*	78	7150	North America	USA	Texas, Hays Co., San Marcos
*Wolffia cylindracea*	180	7340	Africa	Tanzania	Iringa, Mesangati
*Wolffia globosa*	73	9141	South America	Chile	Quillon, Laguna Alendano
*Wolffia neglecta*	624	9149	Asia	Pakistan	Karachi, Gulshan-e-Iasbah
*Wolffiella caudata*	256	9139	South America	Brazil	Amazonas, Manaus
*Wolffiella hyalina*	70	8640	Africa	Tanzania	Arusha, Amboseli
*Wolffiella lingulata*	271	9451	South America	Brazil	not known
*Wolffiella neotropica*	69	7279	South America	Brazil	Rio de Janeiro, Cabo Frio
*Wolffiella oblonga*	46	9136	South America	Brazil	Mato Grosso, Corumba
*Wolffiella repanda*	254	9122	South America	Zimbabwe	Urungwe Safari Area, 12 km SE of Chirundu (from seeds)
*Wolffiella welwitschii*	85	7644	Africa	Angola	Benguela, Cubal

Information from the Rutgers Duckweed Stock Cooperative (http://www.ruduckweed.org/) collection inventory.

**Figure 2:**
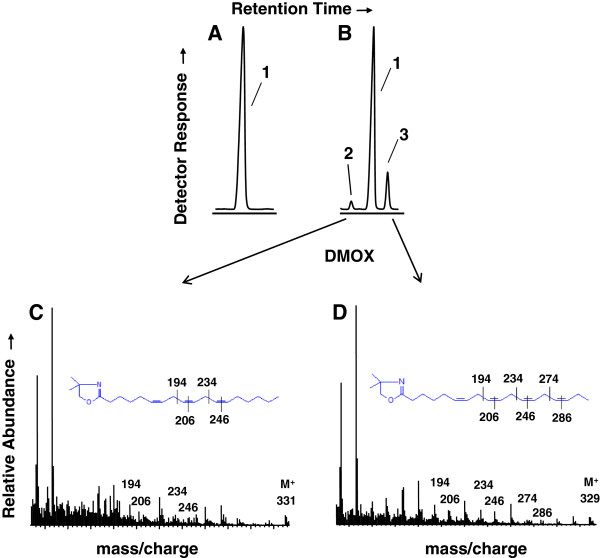
**Identification of GLA and SDA in *Lemna gibba*.** Portions of gas chromatogram traces for FA 4,4-dimethyloxazoline (DMOX) derivatives from *Spirodela polyrhiza* (Panel **A**), and *Lemna gibba* (Panel **B**). Peak labeled 1, corresponds to ALA, 2, GLA, and 3, SDA. Mass spectra corresponding to the DMOX derivatives of GLA (Panel **C**), and SDA (Panel **D**) are shown. Ions diagnostic for the positions of the double bonds in the acyl chains and mass ions are indicated and diagrammed according to W.W. Christie (http://lipidlibrary.aocs.org/ms/masspec.html).

The FAMEs were further derivatized with DMOX to enable diagnostic assignment of double bond positions in FA based on their fragmentation pattern upon mass spectrometric analysis. Three FA, 16:0, 18:2 and 18:3 (Δ9,12,15, or ALA) constitute more than approximately 80% of the FA found in all species (Table 2). 16:0 ranged from 17.2% in *Wolffiella hyalina* to 36.3% in *Wolffiella lingulata*, with a median value of 24.4%; 18:2 ranged from 4.8% in *Wolffiella neotropica* to 25.5% in *Wolffiella borealis* with a median value of 17.5%; and ALA ranged from 34.2% in *Wolffiella borealis* to 64% in *Spirodella polyrhiza* with a median value of 47.7%. In addition to the FA commonly found in terrestrial plants, two unusual species of FA, [M+] ions of m/z 331 and m/z 329, corresponding to 18:3 and 18:4 species, respectively, were identified in a subset of species including *Lemna gibba* but not in others, e.g., *Spirodella polyrhiza* (Figure 2). Mass spectral analysis of the DMOX derivatives of the novel 18:3 species showed it to be GLA (Δ6,9,12,-octadecatrienoic acid) based on fragments corresponding to Δ6 (*m/z* 152, 167 and 180), Δ9 (*m/z* 194, 206) and Δ12 (*m/z* 234, 246) double bonds (Figure 2C). The 18:4 DMOX derivative gave rise to ions corresponding to Δ6,9,12, double bonds as described above for GLA, plus ions characteristic of a Δ15 double bond (*m/z* 274 and 286), identifying it as SDA (18:4Δ6,9,12,15, octadecatetraenoic acid) (Figure 2D). GLA and SDA were detected in six *Lemna* (*L. disperma, L. gibba, L. japonica, L. obscura, L. triscula, L. valdiviana*) and one *Wolffia* (*W. australiana*) species; an additional *Lemna* (*L. valdiviana*) species contained only GLA and a *Wolffia* (*W. cylindracea*) species contained only SDA. The highest level of GLA (4.5%) was identified in *Lemna japonica*; and the highest level of SDA (10.1%) was detected in *Wolffia austrialiana*, which also contained the highest level GLA + SDA combined (11.8%).

**Table 2:** Fatty acid composition as a percentage of total fatty acids of thirty species of duckweed and total fatty acid as a percent of dry weight

**Species**	**Fatty acids (% of total)**											**Total FA /Dry Weight**
	**14:0**	**16:0**	**16:1**	**18:0**	**18:1**	**18:2**	**18:3 (GLA)**	**18:3 (ALA)**	**18:4**	**20:0**	**22:0**	
*Landoltia punctata*	0.3 ± 0.1^1^	24.88 ± 0.3	1.59 ± 0.2	6.57 ± 5.7	0.9 ± 0.4	5.77 ± 1.4	n.d.^2^	60 ± 8.7	n.d.	n.d.	n.d.	7.8 ± 0.6
*Lemna aequinoctialis*	0.6 ± 0.1	26.95 ± 0.6	0.5 ± 0.1	5.51 ± 1.8	1.2 ± 0.2	18.44 ± 0.9	n.d.	45.99 ± 2.0	n.d.	0.3 ± 0.1	0.5 ± 0.4	9.3 ± 1.9
*Lemna disperma*	0.7 ± 0.1	22.1 ± 0.4	2.1 ± 0.4	1.6 ± 0.3	2.3 ± 0.5	12.7 ± 0.2	1.6 ± 0.2	48.9 ± 0.9	7.5 ± 0.1	0.2 ± 0.0	0.3 ± 0.0	11.4 ± 0.9
*Lemna gibba*	0.7 ± 0.2	21.51 ± 0.8	3.62 ± 0.3	3.12 ± 0.8	2.11 ± 0.3	11.06 ± 0.7	1.81 ± 0.2	47.14 ± 0.3	7.74 ± 0.4	0.7 ± 0.0	0.5 ± 0.2	11.2 ± 1.2
*Lemna japonica*	1.1 ± 0.1	18.86 ± 0.2	2.11 ± 0.0	1.5 ± 0.1	2.21 ± 0.0	21.16 ± 0.1	4.51 ± 0.1	40.62 ± 0.0	6.92 ± 0.1	0.4 ± 0.0	0.6 ± 0.1	8.0 ± 0.5
*Lemna minor*	0.55 ± 0.0	21.74 ± 0.5	2.76 ± 0.0	2.1 ± 0.4	1.77 ± 0.1	15.89 ± 0.3	n.d.	54.42 ± 0.7	n.d.	0.33 ± 0.0	0.44 ± 0.1	10.6 ± 0.8
*Lemna minuta*	0.7 ± 0.1	19.32 ± 0.2	1.8 ± 0.0	1.5 ± 0.1	2.2 ± 0.1	15.12 ± 0.3	n.d.	59.06 ± 0.3	n.d.	0.1 ± 0.0	0.2 ± 0.0	7.7 ± 0.1
*Lemna obscura*	0.7 ± 0.1	20.74 ± 0.1	1.4 ± 0.1	2.2 ± 0.2	2.1 ± 0.1	15.03 ± 0.2	1.8 ± 0.1	49.2 ± 0.3	5.61 ± 0.2	0.5 ± 0.1	0.7 ± 0.0	7.5 ± 1.3
*Lemna perpusilla*	0.9 ± 0.1	22.47 ± 0.6	1.2.0.1	2.61 ± 0.5	3.01 ± 0.2	19.56 ± 0.9	n.d.	48.95 ± 2.5	n.d.	0.5 ± 0.0	0.8 ± 0.3	5.5 ± 0.7
*Lemna tenera*	0.7 ± 0.1	28.06 ± 1.3	1 ± 0.1	2.1 ± 0.0	2.51 ± 0.1	17.23 ± 0.2	n.d.	48.4 ± 1.5	n.d.	n.d.	n.d.	6.7 ± 0.6
*Lemna triscula*	1.1 ± 0.1	19.74 ± 0.4	2 ± 0.1	1.7 ± 0.1	2.2 ± 0.0	23.85 ± 0.7	2.3 ± 0.1	43.29 ± 0.1	2.81 ± 0.3	0.5 ± 0.1	0.5 ± 0.0	5.9 ± 0.0
*Lemna turionefera*	1.11 ± 0.1	18.45 ± 0.0	2.22 ± 0.1	1.41 ± 0.1	1.71 ± 0.0	18.75 ± 0.1	3.02 ± 0.5	46.07 ± 0.7	6.35 ± 0.0	0.3 ± 0.0	0.6 ± 0.1	8.3 ± 0.1
*Lemna valdiviana*	0.7 ± 0.0	18.4 ± 0.2	1.2 ± 0.1	1.8 ± 0.2	2.3 ± 0.1	15.8 ± 0.0	0.7 ± 0.4	58.8 ± 0.7	n.d.	0.1 ± 0.0	0.2 ± 0.1	8.0 ± 0.9
*Spirodela intermedia*	0.1 ± 0.0	32.83 ± 5.1	2.2 ± 0.2	3.8 ± 0.7	2.9 ± 0.1	5.21 ± 0.3	n.d.	51.85 ± 5.4	n.d.	0.4 ± 0.1	0.7 ± 0.2	8.4 ± 0.9
*Spirodela polyrhiza*	0.1 ± 0.1	24.44 ± 0.9	1.92 ± 0.5	4.04 ± 3.7	0.2 ± 0.0	4.95 ± 1.5	n.d.	64.04 ± 4.9	n.d.	0.1 ± 0.1	0.2 ± 0.0	7.5 ± 0.2
*Wolffia angusta*	0.6 ± 0.0	24.82 ± 0.1	0.4 ± 0.1	3.7 ± 0.9	3.2 ± 0.1	18.62 ± 0.3	n.d.	47.25 ± 0.8	n.d.	1 ± 0.0	0.4 ± 0.1	8.0 ± 0.9
*Wolffia arrhiza*	0.7 ± 0.4	23.94 ± 2.5	0.8 ± 0.3	2.21 ± 0.2	1.41 ± 0.3	24.45 ± 0.7	n.d.	45.27 ± 3.8	n.d.	0.7 ± 0.1	0.5 ± 0.1	8.9 ± 0.5
*Wolffia australiana*	0.4 ± 0.2	24.22 ± 5.8	2 ± 0.6	2 ± 0.8	1.9 ± 0.5	17.82 ± 0.2	1.7 ± 0.6	38.24 ± 5.5	10.11 ± 2.8	0.5 ± 0.2	1.1 ± 0.5	10.0 ± 0.4
*Wolffia borealis*	0.6 ± 0.2	27.67 ± 1.2	0.9 ± 0.4	7.39 ± 0.9	2.7 ± 1.1	25.47 ± 1.3	n.d.	34.17 ± 2.4	n.d.	1.1 ± 0.3	n.d.	14.2 ± 1.0
*Wolffia brasiliensis*	0.4 ± 0.0	29.55 ± 2.2	1.89 ± 0.0	3.98 ± 0.1	1.99 ± 0.3	18.91 ± 0.1	n.d.	40.3 ± 1.0	n.d.	1.29 ± 0.2	1.69 ± 0.0	5.6 ± 0.5
*Wolffia cylindracea*	0.3 ± 0.1	27.15 ± 1.1	1.8 ± 0.4	4.49 ± 1.7	1.1 ± 0.3	19.56 ± 0.2	n.d.	43.11 ± 2.1	2.1 ± 0.1	n.d.	0.4 ± 0.3	6.7 ± 0.4
*Wolffia globosa*	0.7 ± 0.1	30.36 ± 0.9	n.d.	2.91 ± 0.3	0.5 ± 0.2	14.03 ± 0.8	n.d.	49.2 ± 0.8	n.d.	0.7 ± 0.2	1.6 ± 0.4	11.1 ± 1.0
*Wolffia neglecta*	0.6 ± 0.1	24.53 ± 1.1	0.8 ± 0.3	5.38 ± 0.1	2.89 ± 0.7	24.13 ± 0.9	n.d.	39.98 ± 1.0	n.d.	0.9 ± 0.2	0.8 ± 0.6	12.5 ± 0.8
*Wolffiella caudata*	0.6 ± 0.0	22.65 ± 0.7	0.4 ± 0.0	1.8 ± 0.1	1.9 ± 0.1	24.95 ± 0.4	n.d.	46.11 ± 1.2	n.d.	0.6 ± 0.1	1 ± 0.0	7.0 ± 0.4
*Wolffiella hyalina*	0.3 ± 0.0	17.17 ± 0.4	1.5 ± 0.0	2.2 ± 0.3	3.49 ± 0.1	20.66 ± 0.2	n.d.	51.9 ± 0.9	n.d.	0.8 ± 0.1	2 ± 0.1	10.3 ± 1.2
*Wolffiella lingulata*	0.8 ± 0.1	36.34 ± 4.3	n.d.	7.41 ± 1.1	1.3 ± 0.2	15.82 ± 2.2	n.d.	38.34 ± 2.8	n.d.	n.d.	n.d.	5.7 ± 0.3
*Wolffiella neotropica*	0.6 ± 0.1	27.01 ± 1.6	0.2 ± 0.0	3.92 ± 0.6	0.6 ± 0.1	4.82 ± 0.6	n.d.	61.24 ± 1.9	n.d.	0.4 ± 0.0	1.2 ± 0.1	7.1 ± 0.3
*Wolffiella oblonga*	0.8 ± 0.0	30.16 ± 1.0	0.4 ± 0.0	3.91 ± 0.6	1.3 ± 0.0	11.62 ± 0.8	n.d.	47.09 ± 1.5	n.d.	1.1 ± 0.1	3.61 ± 0.1	10.0 ± 1.4
*Wolffiella repanda*	0.5 ± 0.1	25.45 ± 0.7	1 ± 0.2	2.51 ± 0.6	1.5 ± 0.8	16.03 ± 2.4	n.d.	51.3 ± 0.8	n.d.	0.3 ± 0.1	1.4 ± 0.2	7.6 ± 0.5
*Wolffiella welwitschii*	0.5 ± 0.1	26.65 ± 0.6	0.5 ± 0.2	2 ± 0.4	1.2 ± 0.9	17.84 ± 0.5	n.d.	49.1 ± 1.5	n.d.	0.6 ± 0.1	1.6 ± 0.2	4.6 ± 0.7

^1^Values represent means ± standard deviation of three or more independent samples. ^2^n.d. indicates none detected.

### Identification of a **Δ**6-desaturase from Lemna gibba

**Figure 3:**
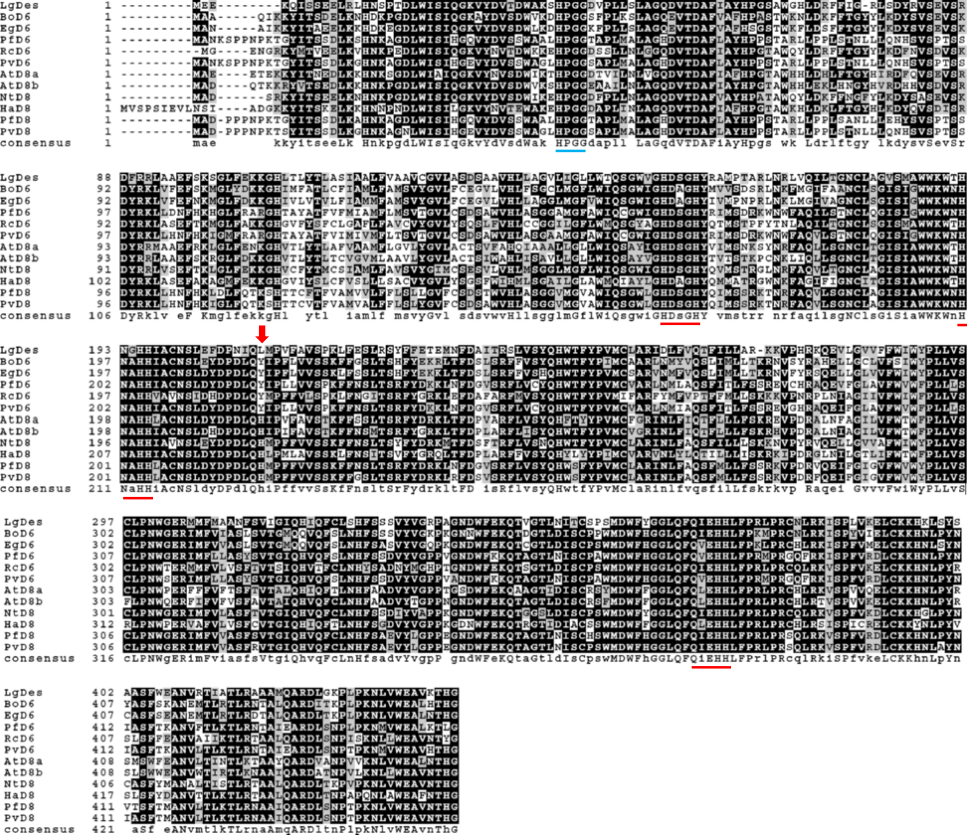
**Sequence comparison of LgDes with experimentally validated homologs.** Alignment was made using CLUSTALW2 [28] and BOXSHADE 3.21. (http://www.ch.embnet.org/software/BOX_form.html). Residues identical for six or more sequences in a given position are in white text on a black background, and for six or more similar residues are white with grey background. Sequences: LgDes (KF638283, this study), Δ6 desaturase sequences: *Borago officinalis* (BoD6, AAC49700) [13]; *Echinum gentianoides* (EgD6, AAL23580) [14], *Primula farinosa* (PfD6, AAP23034) [29], *Ribes nigrum* (RnD6, ADA60230) [30], *Primula vialii* (PvD6, AAP23036) [29]. Δ8 sequences: *Arabidopsis thaliana* (AtD8a, NP_191717; AtD8b, NP_182144) [26], *Nicotiana tabacum* (NtD8, ABO31111) [17], *Helianthus annuus* (HaD8, CAA60621) [31], *Primula farinosa* (PfD8, AAP23033, PvD8, AAP23035) [29]. Three histidine boxes common to membrane bound desaturases are underlined in red. The consensus cytochrome b_5_ sequence is underlined in blue and the amino acid position corresponding to L210 of LgDes, identified by CPDL, is marked with a vertical red arrow.

**Figure 4:**
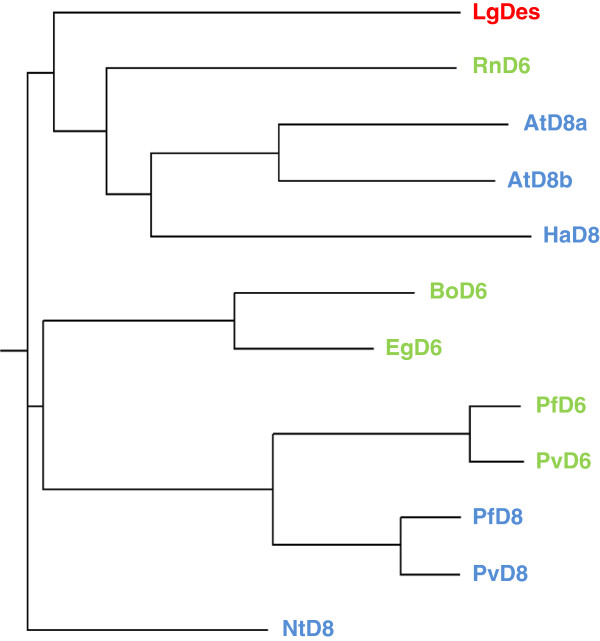
**Phylogenetic tree of LgDes with experimentally validated homologs.** Clustalw2 phylogenetic tree output was fed into the Phylodendron tree drawing tool (http://iubio.bio.indiana.edu/treeapp/treeprint-form.html) to construct the tree by neighbor-joining distance analysis. Line lengths indicate the relative distances between nodes. Sequences and gene identifiers are those described in Figure 3. Color codes: LgDes, red, Δ6-FA desaturases green and Δ8-LCB sphingolipid desaturases, blue.

**Figure 5:**
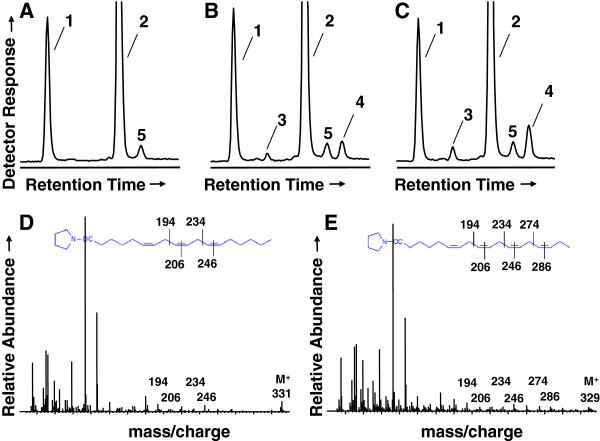
**Expression of LgDes results in the accumulation of GLA and SDA in *N. benthamiana *leaves.** Portions of gas chromatogram traces for FA-pyrrolidide derivatives from *Nicotiana benthamiana* containing empty vector control (Panel **A**), LgDes (Panel **B**), borage Δ6-desaturase (Panel **C**). Peak labeled 1, corresponds to LA, 2, ALA, 3, GLA, 4, SDA, and 5, 20:0. Mass spectra corresponding to the pyrrolidide derivatives of GLA (Panel **D**), and SDA (Panel **E**) derived from the expression of LgDes shown in Panel B, peaks 3 and 4, respectively, are shown. Ions diagnostic for the positions of the double bonds in the acyl chains and the mass ions are indicated and diagrammed according to W.W. Christie (http://lipidlibrary.aocs.org/ms/masspec.html).

We hypothesized that Δ6-desaturation in the Lemnoideae might result from the action of a front-end desaturase [15] with similarity to those of higher land plants. We began our investigation by performing a TBLASTN query against our *de novo* assembled transcript database of *Lemna gibba* G3 DWC131 (unpublished, http://www.lemna.org) with the amino acid sequence of *Borago officinalis* Δ6-desaturase, Boofd6 [13], which is responsible for the synthesis of GLA from LA and SDA from ALA. The search returned a candidate sequence we designated LgDes (Genbank accession: KF638283) that shares 62% amino acid identity to the *Borago officinalis* Δ6-desaturase (Table 3). A reciprocal search of LgDes against GenBank identified many front-end desaturase homologs that were annotated either as Δ6-FA desaturases or Δ8-sphingolipid long-chain-base (LCB) desaturases. We narrowed our search to include only genes for which annotation was supported by functional expression experiments. This yielded five Δ6-FA desaturases and six Δ8-LCB desaturases (see Figure 3). Multiple sequence alignment of LgDes with these sequences reveals blocks of strong homology including the regions around the three histidine clusters characteristic of membrane desaturases [16]. Attempting to distinguish whether LgDes encodes a Δ6-FA desaturases or Δ8-LCB desaturase, we performed pairwise comparisons between the LgDes and the 11 experimentally defined sequences (Table 3). The closest homolog of LgDes was the tobacco Δ8-LCB desaturase (70%) [17] followed by Δ6-FA desaturases from *Echinum gentianoides* (65%) and *Ribes nigrum* (63%), an *Arabidopsis* Δ8-LCB desaturase [26] and the borage Δ6-FA desaturase [13] (both at 62%). These relationships can be visualized as a phylogenetic tree (Figure 4) and it is striking that the Δ6-FA desaturases or Δ8-LCB desaturases don’t cluster in distinct clades. Because LgDes does not fall within a discrete clade of Δ6-FA or Δ8-LCB desaturases, we assessed the function of the LgDes by heterologous expression and phenotypic analysis. To achieve this, LgDes and the borage Δ6-FA desaturase were cloned into binary plant expression vectors that were used for agrobacterium-mediated transient expression in *Nicotiana benthamiana* leaves [27]. A control consisting of empty vector was used as a negative control. Four days after inoculation, leaf FA were extracted and analysed by mass spectrometry. As shown in Figure 5 panels A-C, peaks eluting with mobilities consistent with GLA and SDA standards were detected in leaf samples transformed with the *Borago officinalis* Δ6-desaturase and LgDes, but not with the negative control plasmid. Mass spectra corresponding to GLA and SDA pyrrolidide derivatives confirmed the identity of these FA (Figure 5, D and E). Together, these data conclusively establish that LgDes acts as a Δ6-FA desaturase thought the current analysis cannot preclude the formal possibility that it can also perform Δ8-LCB sphingolipid desaturation. The conversion efficiency of LgDes under our experimental conditions was approximately half that of the borage desaturase BoD6 (Table 4). LgDes converted approximately 4% of LA to GLA and 3% of ALA to SDA.

**Table 3:** Amino acid identities computed using the ClustalW2 multiple sequence alignment algorithm

	**LgDes**	**BoD6**	**EgD6**	**PfD6**	**RnD6**	**PvD6**	**AtD8a**	**AtD8b**	**NtD8**	**HaD8**	**PfD8**	**PvD8**
LgDes												
BoD6	62											
EgD6	65	85										
PfD6	58	65	65									
RnD6	63	61	64	58								
PvD6	57	64	63	95	57							
AtD8a	61	58	62	58	65	57						
AtD8b	62	60	61	58	66	59	80					
NtD8	70	73	73	64	71	64	66	66				
HaD8	59	61	61	56	65	56	67	67	64			
PfD8	61	61	63	80	61	80	62	63	71	59		
PvD8	61	62	64	80	62	81	62	63	71	59	94	

**Table 4:** **Conversion efficiencies of desaturases expressed in *Nicotiana benthamiana***

**Desaturase**	**Conversion**	**Conversion %**
**LgDes**	**18:2 to 18:3**	**3.76 ± 0.04**^**1**^
	**18:3 to 18:4**	**3.28 ± 1.27**
**BoD6**	**18:2 to 18:3**	**6.89 ± 1.17**
	**18:3 to 18:4**	**6.07 ± 2.73**

^1^Values represent means ± standard deviation of three or more independent samples.

### Triacyl Glycerol content and composition

TAG from each duckweed species was identified based on co-migration with an Arabidopsis TAG standard with the use of thin-layer chromatography (TLC). The region of TLC plates corresponding to TAG was collected, extracted and the FA converted to their corresponding FAMEs which were subsequently separated by GC/MS. The TAG content of each species (Table 5) ranges from 0.02% in *Spirodela polyrhiza* to 0.15% in *Wolffiella lingulata* with a median value of 0.06%. The FA composition of TAG is similar to that of the total FA in that more than 80% of the FA consists of 16:0, 18:0, 18:2 and 18:3 (ALA). However, total FA contain 28% saturates (16:0 + 18:0) and 64% polyunsaturates (18:2 and 18:3 ALA), whereas this pattern is reversed in TAG which contains 57% saturates and only 33% polyunsaturates. Thus, both saturates, 16:0 and 18:0 are enriched in TAG relative to total FA (40% *vs*. 25% and 17% *vs*. 3%, respectively), 18:2 is essentially unchanged (15% *vs.* 16%) and 18:3 ALA is strongly reduced in TAG relative to total FA (18% *vs.* 48%).

**Table 5:** Fatty acid composition of TAG as a percentage of total TAG, and TAG as a percentage of total lipid or dry weight

	**Fatty acids (% of total)**											**% TAG per mg of lipid**	**% TAG per mg dry weight**
	**14:0**	**16:0**	**16:1**	**18:0**	**18:1**	**18:2**	**18:3 (GLA)**	**18:3 (ALA)**	**18:4**	**20:0**	**22:0**		
*Landoltia punctata*	0.6 ± 0.2^1^	39.6 ± 9.4	n.d. ^2^	5.4 ± 0.6	6.7 ± 1.4	12.2 ± 1.2	n.d.	35.5 ± 7.9	n.d.	n.d.	n.d.	0.7 ± 0.1	0.06 ± 0.01
*Lemna aequinoctialis*	2 ± 0.5	36.1 ± 1.9	n.d.	28.4 ± 1.8	3.8 ± 1.2	11.9 ± 1.6	n.d.	17.8 ± 3	n.d.	n.d.	n.d.	0.6 ± 0.1	0.06 ± 0.01
*Lemna disperma*	2.7 ± 0.4	44 ± 12.6	n.d.	22.1 ± 10.2	7.3 ± 7.3	10.4 ± 1.7	n.d.	13.4 ± 3.1	n.d.	n.d.	n.d.	0.5 ± 0.1	0.05 ± 0.02
*Lemna gibba*	0.3 ± 0.1	30.5 ± 2.3	n.d.	6.2 ± 0.6	7.1 ± 1.8	17 ± 1.3	n.d.	33 ± 2.3	5.9 ± 1.1	n.d.	n.d.	0.5 ± 0.1	0.06 ± 0.00
*Lemna japonica*	1.9 ± 0.7	36.2 ± 1.7	n.d.	6.6 ± 0.6	3.4 ± 0.1	22.9 ± 0.9	5.5 ± 0.5	18.5 ± 0.5	5.1 ± 0.1	n.d.	n.d.	0.9 ± 0.0	0.08 ± 0.01
*Lemna minor*	2.5 ± 0.4	44 ± 1.9	n.d.	15 ± 0.5	4.1 ± 1.0	12.5 ± 1.9	n.d.	22 ± 0.9	n.d.	n.d.	n.d.	0.3 ± 0.0	0.03 ± 0.01
*Lemna minuta*	1.1 ± 0.1	38.4 ± 0.5	n.d.	11.1 ± 0.5	6.9 ± 0.1	16.3 ± 0.7	n.d.	26.2 ± 1.1	n.d.	n.d.	n.d.	0.8 ± 0.1	0.06 ± 0.01
*Lemna obscura*	1.7 ± 0.2	29.2 ± 4.2	n.d.	16.1 ± 1.6	3.8 ± 1.2	13.7 ± 1.6	n.d.	31.5 ± 6.1	3.9 ± 0.9	n.d.	n.d.	0.7 ± 0.2	0.05 ± 0.00
*Lemna perpusilla*	0.8 ± 0.0	35.2 ± 0.2	n.d.	8.4 ± 1.3	5.2 ± 0.5	23.3 ± 0.0	n.d.	23.8 ± 3.2	n.d.	1.6 ± 0.2	1.6 ± 0.9	1.4 ± 0.2	0.08 ± 0.01
*Lemna tenera*	1.2 ± 0.1	46.7 ± 1.1	n.d.	17 ± 3.5	5.9 ± 0.1	12.3 ± 1.1	n.d.	17 ± 2.9	n.d.	n.d.	n.d.	0.9 ± 0.2	0.06 ± 0.01
*Lemna triscula*	1.1 ± 0.1	20.7 ± 1.3	n.d.	7 ± 1.3	1.8 ± 0.1	32.7 ± 0.5	3.3 ± 0.7	33.5 ± 0.6	n.d.	n.d.	n.d.	0.6 ± 0.1	0.04 ± 0.01
*Lemna turionefera*	1.6 ± 0.3	30.5 ± 2.2	n.d.	9.4 ± 0.2	2.5 ± 0.7	20.3 ± 2.7	5.8 ± 0.1	25.9 ± 1.4	4.1 ± 1.1	n.d.	n.d.	0.6 ± 0.1	0.05 ± 0.01
*Lemna valdiviana*	1.3 ± 0.0	36.3 ± 2.2	n.d.	13 ± 2.0	5.4 ± 0.4	15.7 ± 0.9	n.d.	28.4 ± 2.3	n.d.	n.d.	n.d.	0.6 ± 0.1	0.05 ± 0.01
*Spirodela intermedia*	2 ± 0.6	41.8 ± 2.1	n.d.	22.7 ± 3.1	3.3 ± 1.3	4.7 ± 1.3	n.d.	25.6 ± 1.7	n.d.	n.d.	n.d.	0.4 ± 0.1	0.03 ± 0.00
*Spirodela polyrhiza*	1.6 ± 0.3	42.4 ± 3.9	n.d.	12.2 ± 4.1	3.7 ± 0.9	10.1 ± 1.0	n.d.	29.9 ± 0.1	n.d.	n.d.	n.d.	0.2 ± 0.0	0.02 ± 0.00
*Wolffia angusta*	1.9 ± 0.1	34.2 ± 1.2	n.d.	14.2 ± 0.4	9.80.02	29.6 ± 0.7	n.d.	10.3 ± 0.8	n.d.	n.d.	n.d.	1.3 ± 0.2	0.1 ± 0.01
*Wolffia arrhiza*	5.3 ± 0.3	33.9 ± 2.9	n.d.	17.4 ± 0.8	6.8 ± 2.0	23.7 ± 1.0	n.d.	12.9 ± 1.7	n.d.	n.d.	n.d.	0.6 ± 0.1	0.05 ± 0.00
*Wolffia australiana*	2.5 ± 0.5	38.1 ± 5.5	n.d.	24.3 ± 2.7	7.4 ± 2.7	17 ± 3.7	n.d.	10.8 ± 1.9	n.d.	n.d.	n.d.	0.2 ± 0.1	0.02 ± 0.01
*Wolffia borealis*	6.4 ± 0.4	38.5 ± 3.4	n.d.	34.5 ± 3.0	n.d.	9 ± 4.3	n.d.	11.7 ± 2.9	n.d.	n.d.	n.d.	0.3 ± 0.1	0.04 ± 0.02
*Wolffia brasiliensis*	1.3 ± 0.0	46.1 ± 0.3	n.d.	32 ± 1.5	3.7 ± 0.0	8 ± 0.1	n.d.	8.9 ± 0.2	n.d.	n.d.	n.d.	0.8 ± 0.1	0.04 ± 0.00
*Wolffia cylindracea*	1.1 ± 0.2	54.7 ± 1.0	n.d.	7 ± 0.7	4.5 ± 0.9	24 ± 0.8	n.d.	8.7 ± 1.7	n.d.	n.d.	n.d.	1.0 ± 0.3	0.07 ± 0.02
*Wolffia globosa*	2.5 ± 0.7	43.8 ± 2.4	n.d.	25.2 ± 4.8	6.5 ± 6.1	9.3 ± 2.2	n.d.	12.6 ± 6.2	n.d.	n.d.	n.d.	0.6 ± 0.3	0.06 ± 0.03
*Wolffia neglecta*	4.5 ± 0.9	48.5 ± 1.5	n.d.	29.1 ± 0.3	4.9 ± 1.2	6.6 ± 1.5	n.d.	6.5 ± 1.3	n.d.	n.d.	n.d.	0.3 ± 0.0	0.04 ± 0.00
*Wolffiella caudata*	1.6 ± 0.1	51.2 ± 1.3	n.d.	9.8 ± 1.5	3 ± 0.3	17.1 ± 0.1	n.d.	17.3 ± 1.6	n.d.	n.d.	n.d.	0.7 ± 0.1	0.05 ± 0.01
*Wolffiella hyalina*	2.1 ± 0.6	47.1 ± 3.2	n.d.	15.7 ± 1.4	9.8 ± 1.6	14.5 ± 2.0	n.d.	10.9 ± 1.4	n.d.	n.d.	n.d.	0.6 ± 0.1	0.06 ± 0.01
*Wolffiella lingulata*	1.8 ± 0.2	47 ± 2.1	n.d.	32.3 ± 3.7	3.8 ± 0.7	5.9 ± 0.7	n.d.	9.2 ± 2.7	n.d.	n.d.	n.d.	2.7 ± 0.3	0.15 ± 0.02
*Wolffiella neotropica*	1.9 ± 0.4	47.2 ± 2.7	n.d.	23.8 ± 5.0	5.1 ± 4.6	7.1 ± 3.7	n.d.	14.9 ± 3.7	n.d.	n.d.	n.d.	0.9 ± 0.1	0.06 ± 0.01
*Wolffiella oblonga*	2.4 ± 0.1	39.3 ± 2.5	n.d.	24.8 ± 2.6	4.9 ± 3.5	12.7 ± 5.5	n.d.	15.9 ± 4.1	n.d.	n.d.	n.d.	1.1 ± 0.2	0.11 ± 0.00
*Wolffiella repanda*	1.7 ± 0.5	48.8 ± 4.9	n.d.	11.5 ± 1.3	3.5 ± 1.1	17.1 ± 1.5	n.d.	17.4 ± 2.9	n.d.	n.d.	n.d.	1.0 ± 0.1	0.07 ± 0.00
*Wolffiella welwitschii*	0.7 ± 0.3	52.9 ± 1.2	n.d.	19.3 ± 9.6	2.5 ± 0.6	14.3 ± 5.3	n.d.	10.3 ± 4.2	n.d.	n.d.	n.d.	0.9 ± 0.4	0.04 ± 0.01

^1^Values represent means ± standard deviation of three or more independent samples. ^2^n.d. indicates none detected.

Whereas nine duckweed species accumulate Δ6-containing FA, only five *Lemna* species (*L. gibba, L. japonica, L. obscura, L. triscula, L. turionefera*) accumulated them in TAG. In the three species (*L. japonica, L. triscula, L. turionefera*) that accumulated GLA in TAG, levels were significantly higher (student T-test, p < 0.05) by approximately 50% in TAG than in the total FA whereas SDA was significantly decreased (student T-test, p < 0.05) in TAG relative to total FA by approximately 50%. Only two of the nine species of duckweed (*L. japonica, L. turionefera*) contain both GLA and SDA in both their total FA and TAG (Table 5).

## Discussion

This study represents the most comprehensive survey of both FA and TAG composition and content of duckweed undertaken to date with the 30 species chosen to represent the range of diversity of Lemnoideae family with respect to both morphology and geographic origins.

The FA content shows a 3-fold range; *Wolffia borealis* having the highest at 14.2% of dry weight with *Wolffiella welwitschii* at 4.6%. Total FA profiles show little variation with ALA and palmitic acid together accounting for >60%. All the surveyed species have similar FA compositions within their TAG in which > 50% of the FA is composed of palmitic acid plus ALA. Stearic acid represents >10% of FA in the TAG in 22 of the species surveyed.

While the total FA compositions and the TAG FA compositions are similar across the Lemnoideae, the total FA composition differs from that of TAG. The level of saturated FA in TAG is approximately twice that of the total FA pool, with ALA decreasing to compensate; specifically stearic acid comprises <7% of the total FA, but represents 10-34% of TAG in 22 of the duckweed species. The Δ6-double bond–containing FA SDA follows the same pattern, its levels being 51% lower in TAG relative to the total FA pool, whereas GLA, like saturated FAs, is a preferred substrate for TAG incorporation in the three *Lemna* species*, japonica, triscula* and *turionefera*, with its levels increasing by 50% in TAG relative to the total FA pool. The increase in saturates and GLA in TAG relative to the total FA pool implies that these three duckweed species contain acyltransferases with preference for saturated FA and GLA. An alternate explanation is that each duckweed species has several acyl transferases that show differential selectivity with respect to saturated (and GLA), and polyunsaturated FA substrates which are expressed at different levels in different duckweed species. These results provide information that may be helpful to identify duckweed species that contain TAG biased towards desired FA compositions.

The fronds of duckweeds have much lower oil content than the seeds of soybean, rapeseed, cottonseed and peanut, the world’s major oil crops. *Wolffiella lingulata* exhibits the highest oil content at 0.14% of total dry mass, which is 200-fold lower than that of soybean seed [32] and 400 times lower than that of sunflower seed [33]. This large difference is mainly due to the fact that duckweed harvested for this study comprises vegetative tissue, and a more appropriate comparison would be to that of other vegetative tissues. For instance, Arabidopsis leaves contain approximately 0.06% of dry mass as TAG [8], crabapple leaves contains approximately 0.15% TAG and *L. serriola* leaves contain around 0.5% TAG [34]. Thus, *Wolffiella lingulata’s* TAG content of 0.15% falls within the range reported for terrestrial vegetative tissues.

**Figure 6:**
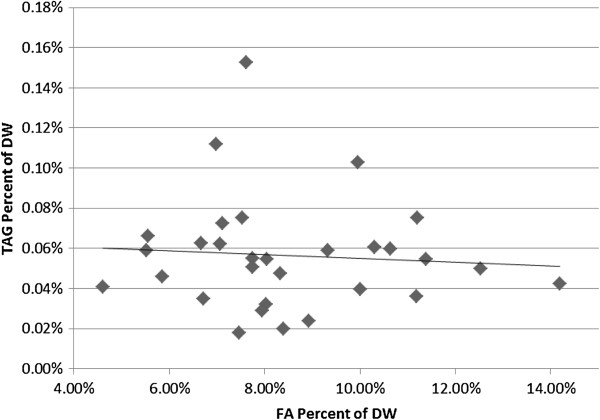
Scatter plot of TAG per DW as a function of total FA per DW.

That the total FA content varies by more than three-fold and TAG content varies by approximately 7-fold within the 30 duckweed species prompted us to ask whether there is a relationship between TAG accumulation and total FA accumulation levels. Significant variation of approximately 14-fold is observed in the TAG/total FA ratio, with *Wolffia austrialiana* having the lowest ratio at 0.2% and *Wolffiella lingulata* having the highest ratio at 2.7%. However, *Wolffiella lingulata* could be considered an outlier because its ratio is almost double that of the next highest ranked species, *Lemna perpussila* at 1.4%. If the outlier is omitted, the range is closer to 7-fold, i.e., in a similar range to that of total FA and TAG contents. This observation prompted us to ask whether TAG content, which is a very small proportion (<0.8% of the total FA), is dependent on the level of total FA. We therefore made a scatter plot of TAG content versus total FA content of the 30 duckweed species (Figure 6). TAG accumulation is not significantly correlated with the level of total FA accumulation (*R*^2^ < 0.1). Indeed, the highest and lowest TAG contents are found in species that accumulate approximately 7.5% of total FA, i.e., close to the median total FA content of 8%, and the species containing the lowest and highest total FA content both accumulate only 0.04% TAG. That the proportion of FA that accumulate in TAG is very low (<0.8%) compared to total FA suggests that TAG assembly is limiting in duckweed tissue, and that increasing the expression of acyltransferases along with oil-body structural proteins such as oleosins and caleosins might represent attractive targets for increasing TAG accumulation [11,35]. At a point where TAG starts to deplete the total FA pool to a significant degree, further enhancements in TAG accumulation could be envisaged by increasing the rate of FA biosynthesis, by ectopic expression of transcription factors such as wrinkled 1 (WRI1) and by inhibiting starch biosynthesis [10]. Increasing FAS could also be achieved by overexpressing other transcription factors known to enhance FA biosynthesis such as LEC2 and FUS3 [36], or by strategies to increase the activity of the first committed step in FA biosynthesis acetyl co-A carboxylase [37].

Lipid composition is relatively uniform across all the surveyed duckweeds, with the exception of the presence of Δ6-FA in nine duckweed species. GLA and SDA are uncommon, having being found only in a small number of higher plants [38], including borage [13] and *Echium*[14]. Our finding that the *Lemna gibba* Δ6 desaturase LgDes showed stronger homology to a tobacco Δ8-shingolipid desaturase [17] than to the borage Δ6 desaturase, which was used as a homology probe to identify LgDes, supports the observation that Δ6-FA desaturases and Δ8-LCB sphingolipid desaturases are closely related and likely have evolved from each other a number of times during evolution [15]. It is interesting in this regard that expression experiments using closely related Δ6-FA and Δ8-LCB desaturases in yeast showed them to have non-overlapping functions [39]. The availability of both substrates in yeast suggests that the context of the acyl chain as a FA or LCB does not influence regioselectivity for these enzymes as it does for a family of ADS enzymes in which the nature of the head group determines whether a Δ7 or Δ9 double bond is introduced to the 16:0 FA substrate [40]. It would be interesting to determine whether duckweed species that lack detectable Δ6-FA also lack the Δ6-desaturase gene, or whether it is present but not expressed under our growth conditions. The traditional approach of southern or northern blotting is not useful in this case because of the generally high sequence similarity between Δ6-FA and Δ8-LCB desaturase gene sequences (Figures 3 and 4 and [15]). We therefore searched the unpublished genomes of a Δ6-FA-containing species *Lemna gibba*, and the Δ6-FA-lacking species, *Spirodela polyrhiza* for sequences homologous to LgDes. One putative desaturase was identified in each genome, which showed higher homology to each other than to LgDes, suggesting they encode Δ8-LCB desaturases. Our search therefore failed to provide evidence for a *Spirodela polyrhiza* Δ6-FA desaturase with close homology to LgDes.

In an attempt to identify locations that might help explain the difference in enzyme specificity between Δ6-FA and six Δ8-LCB desaturases we performed conserved property difference locator (CPDL) analysis [41] using six functionally enzymes from each class (Additional file 1: CPDL analysis of six D6 and six D8 desaturases). Many amino acid locations were identified as potential specificity determining residues however, only one location (at amino acid 210), showed a consistent property difference between the Δ6-FA and Δ8-LCB class of desaturases (see Figure 3, vertical red arrow). The position occupied by the neutral amino acids Leu210, in LgDes, and Tyr in the other five Δ6 desaturases is occupied by a charged His residue in all six Δ8 desaturase sequences. The hypothesis that the nature of the amino acid at position 210 along with other factor(s) that lie between amino acids 80 and 350 specify Δ6 FA or Δ8 LCB activity is consistent with the available borage chimera data [39].

## Conclusions

The survey of 30 species duckweeds showed total FA content varied approximately 3-fold between 4.6% and 14.2% of dry weight; however, there is surprisingly low variability in FA composition between the 30 duckweed species surveyed, with three FA, palmitic, LN and ALA comprising more than 80% of total duckweed FA. However, the Δ6-FA GLA and/or SDA were identified in seven *Lemna* and two *Wolffiela* species. LgDes, a desaturase, with homology to a family of front-end Δ6-FA and Δ8-spingolipid desaturases, comprising a cytochrome b_5_-desaturase fusion, was isolated from *Lemna gibba* and functionally confirmed as a Δ6-FA desaturase by expression in *Nicotiana benthamiana*. In duckweed, TAG enriched in saturated FA at the expense of polyunsaturated FA accumulates at up to 0.15% of total FA, i.e., at levels comparable to those found in the leaves of terrestrial plants. Δ6-FA occur in the TAG of only five *Lemna* species, within which GLA is enriched, and SDA diminished, relative to their abundance in the total FA pool.

## Competing interests

The authors declare no financial or other competing interests.

## Authors’ contributions

JS conceived of and provided the initial design of the study. YY JC, HS and EE performed the research; EE and RM created the *Lemna gibba* transcript sequence and assembly. All authors contributed to data analysis and manuscript preparation. All authors have read and approved the final manuscript.

## Supplementary Material

Additional file 1 CPDL analysis of six D6 and six D8 desaturases. Click here for file
