# Supplementary material for: Survey of the total fatty acid and triacylglycerol composition and content of 30 duckweed species and cloning of a Δ6-desaturase responsible for the production of γ-linolenic and stearidonic acids in Lemna gibba
Source: BMC Plant Biol. 2013 Dec 5;13:201. doi: 10.1186/1471-2229-13-201 (PMC3879013; doi:10.1186/1471-2229-13-201)
Supplement: Additional file 1 — CPDL analysis of six D6 and six D8 desaturases. [file 1471-2229-13-201-S1.pdf]

CPDL analysis of six D6 and six D8 desaturases

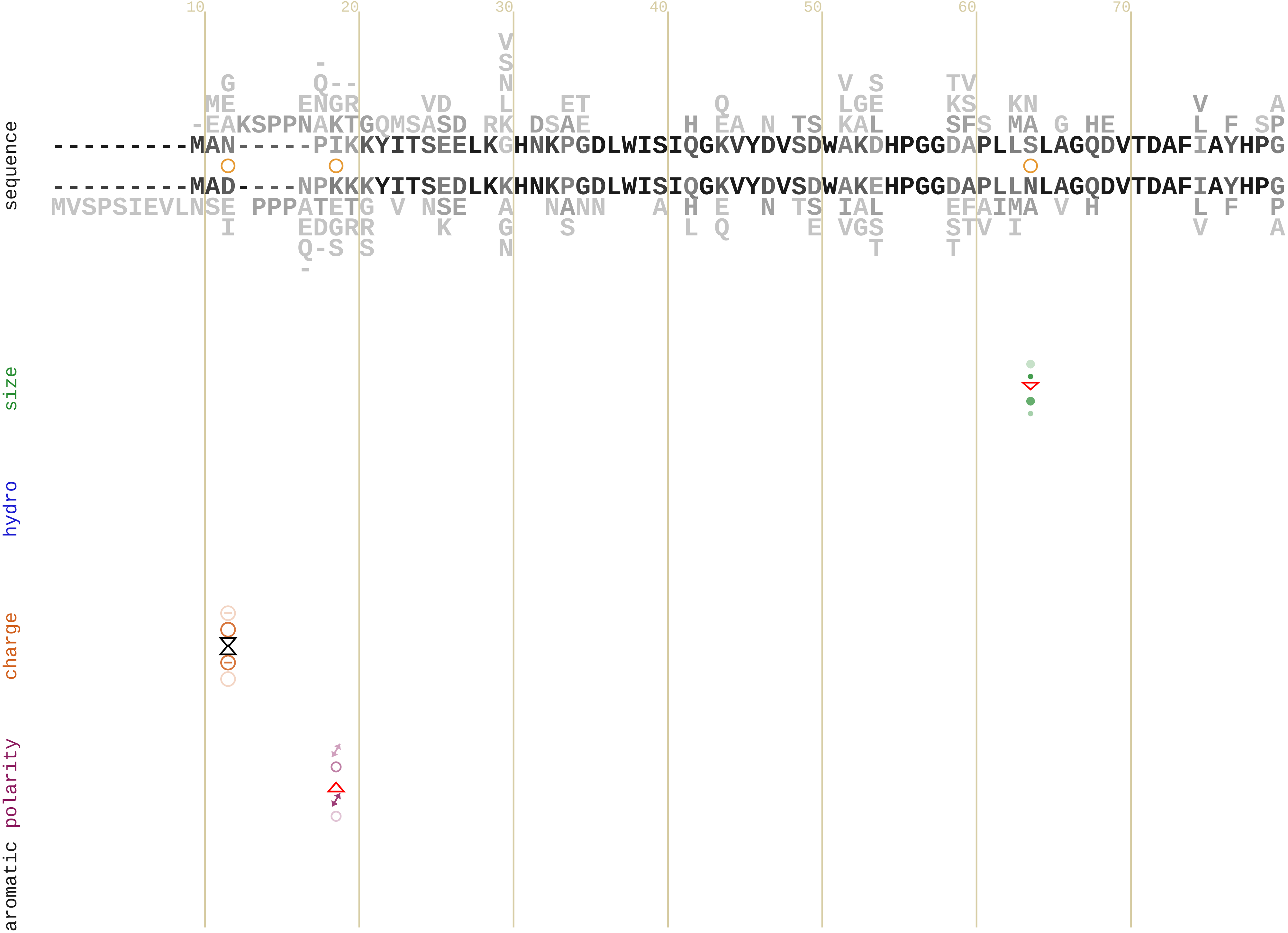

CPDL analysis of six D6 and six D8 desaturases

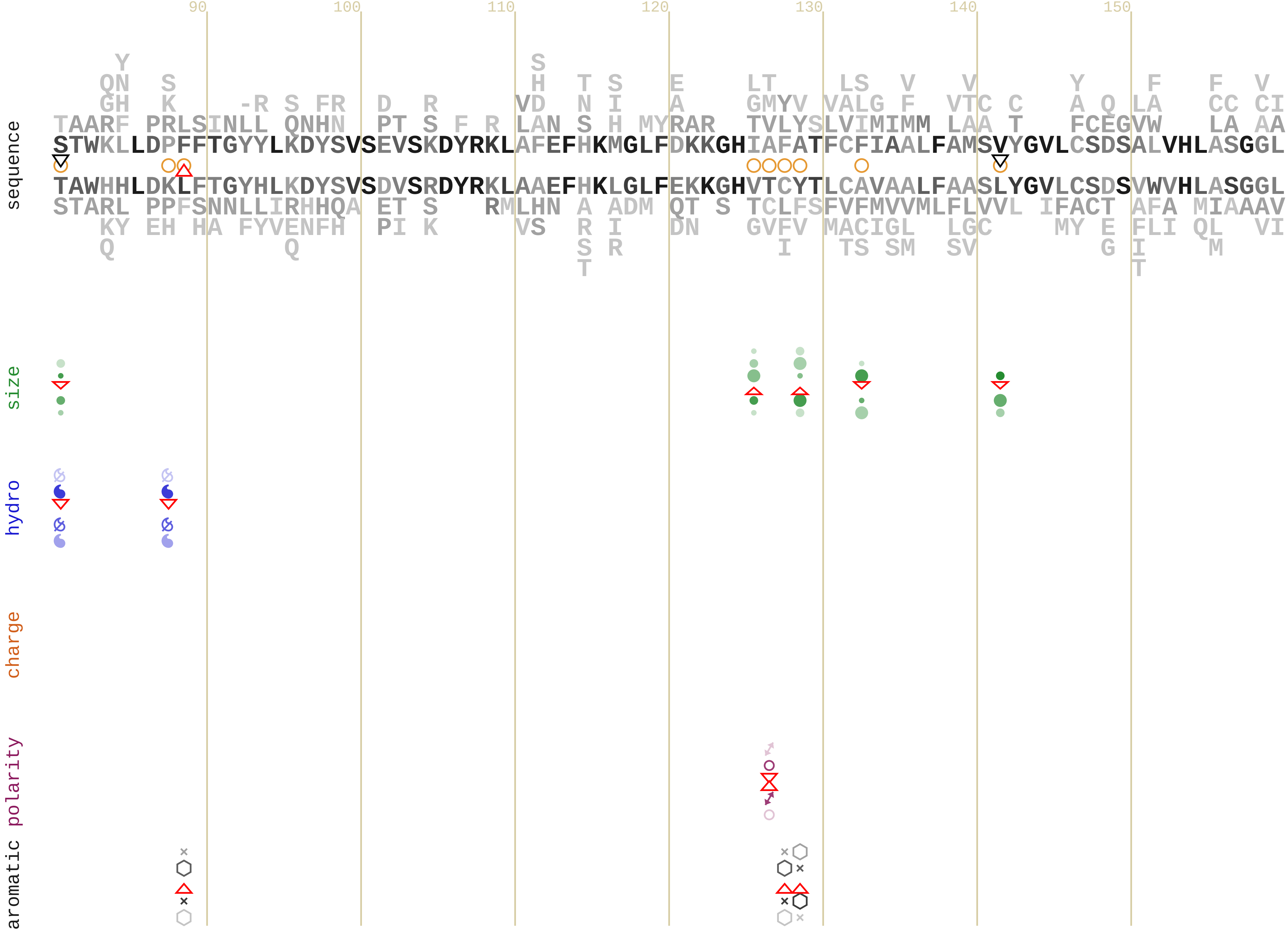

CPDL analysis of six D6 and six D8 desaturases

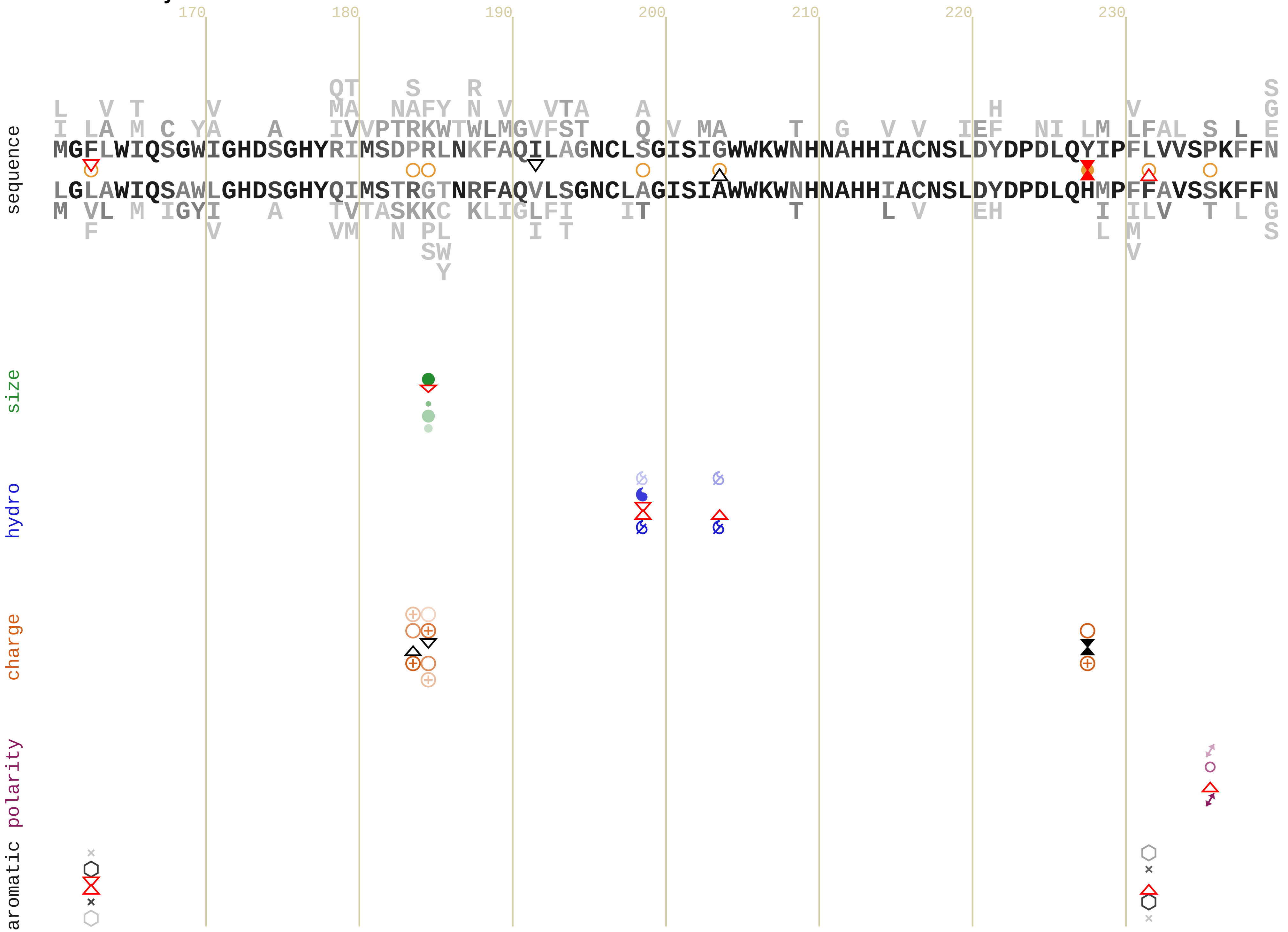

CPDL analysis of six D6 and six D8 desaturases

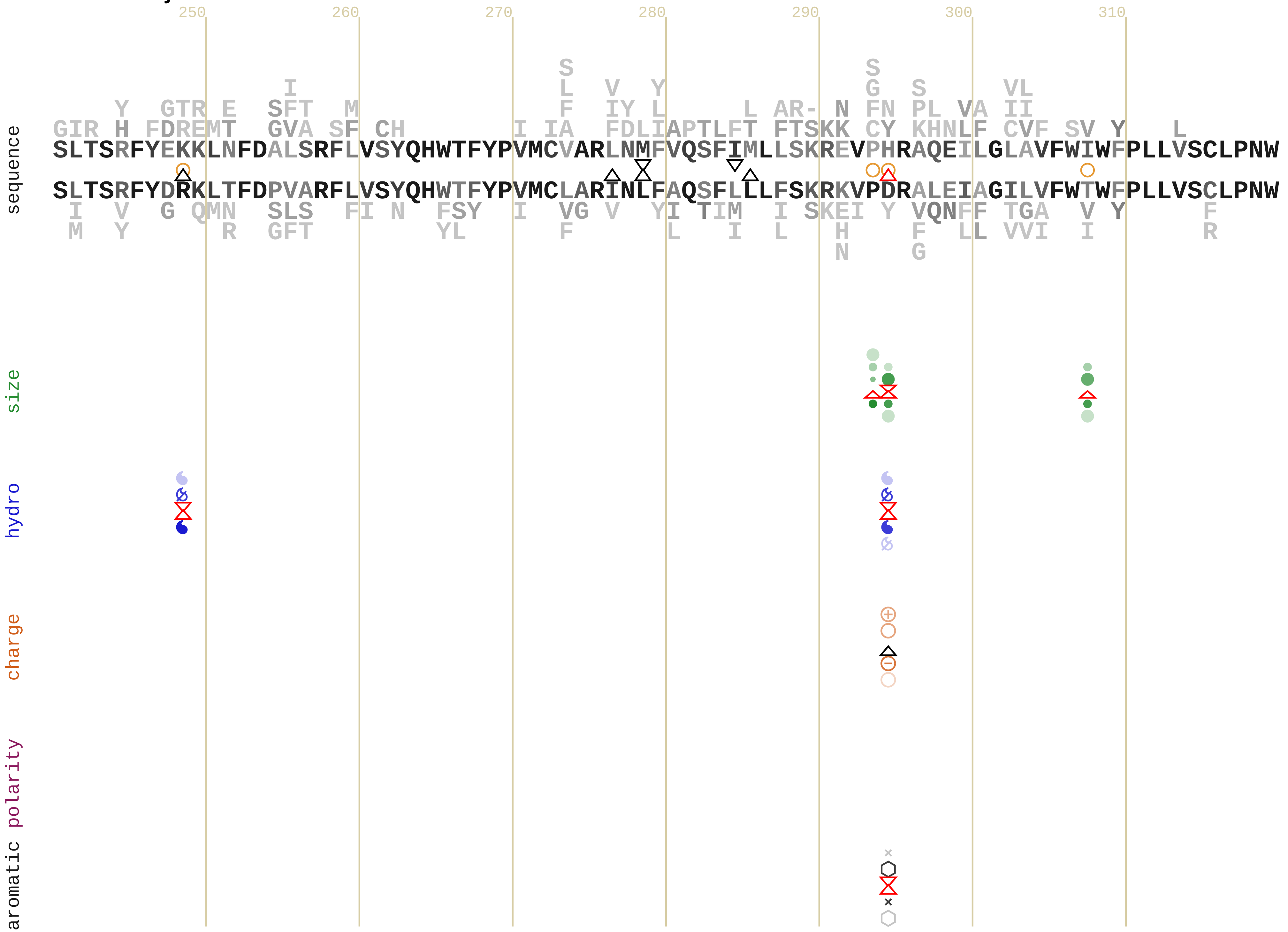

CPDL analysis of six D6 and six D8 desaturases

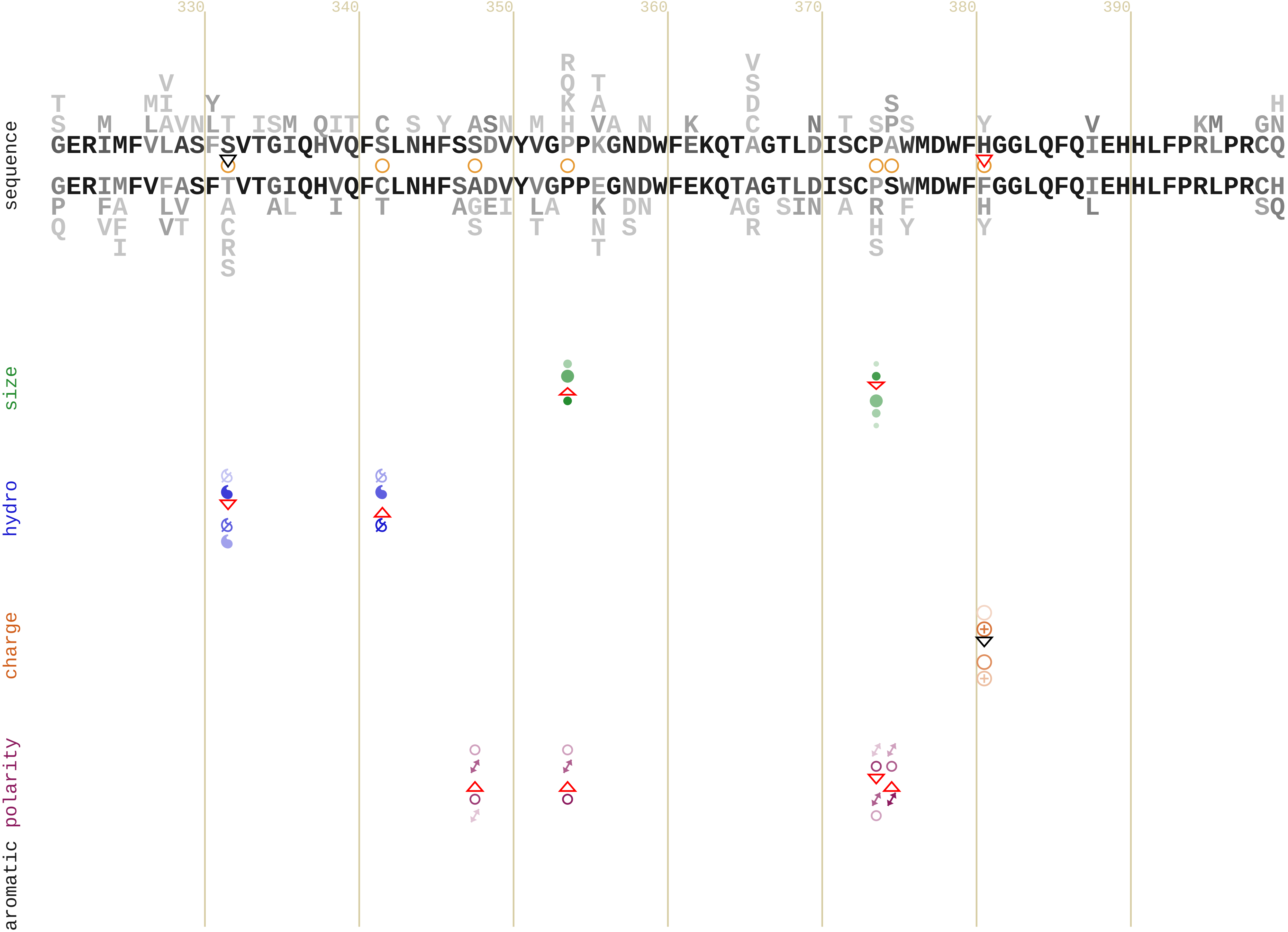

CPDL analysis of six D6 and six D8 desaturases

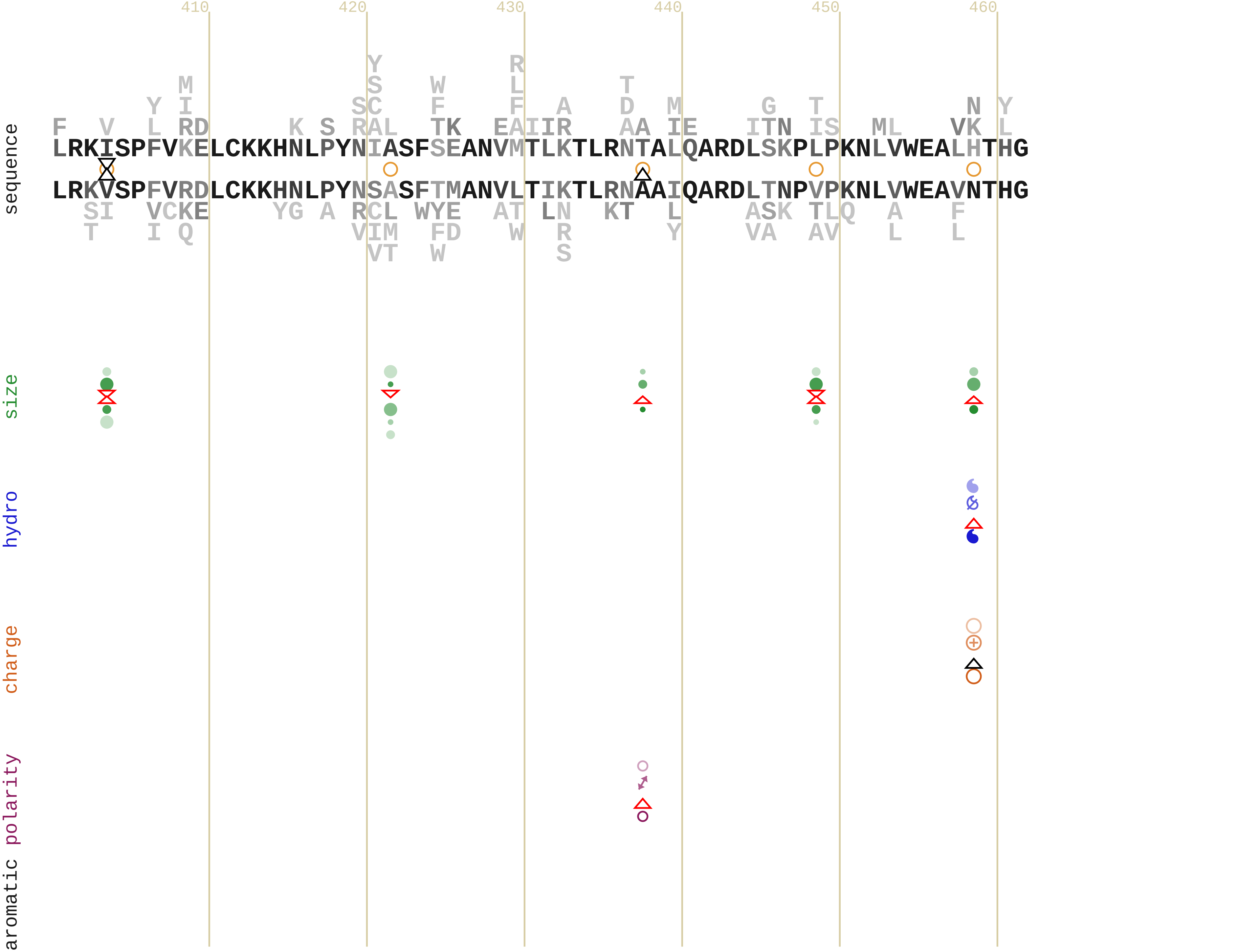

Residue Properties/Symbol Key

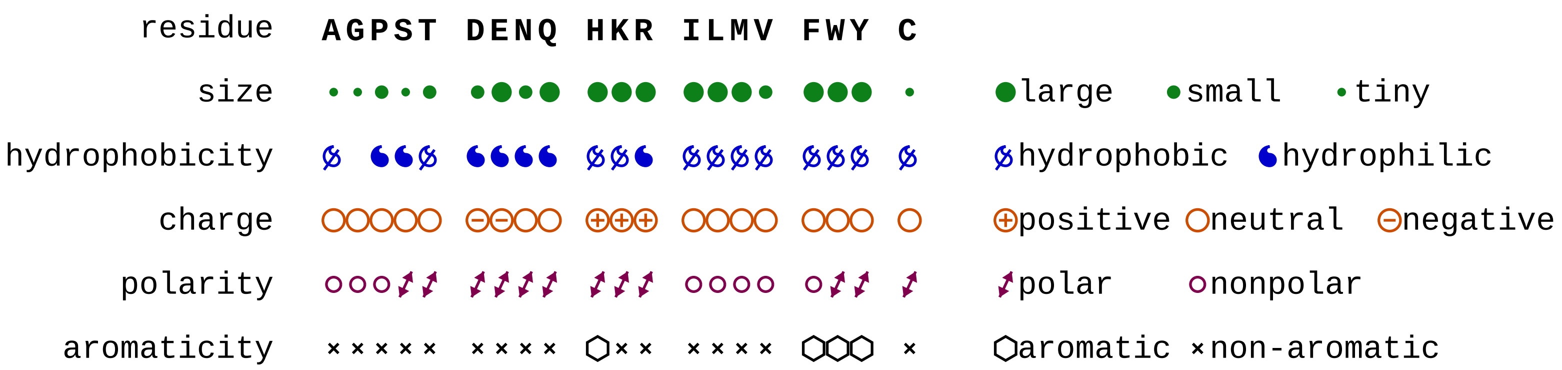

User settings:

Alignment file: twelve_seqs_from_john_and_jorg.aln
Top group rows 1-6; Bottom group rows 7-12
Property track display settings:
   size: if in track
   hydro: if in track
   charge: if in track
   polarity: if in track
   aromatic: if in track
Conservation level: all or all but one
Flag conserved residue property differences in main track with orange circles
Grayscale function: linear
Darkness adjustment: 0.25
